# Exploring Marine Planktonic Archaea: Then and Now

**DOI:** 10.3389/fmicb.2020.616086

**Published:** 2021-01-13

**Authors:** Edward F. DeLong

**Affiliations:** Daniel K. Inouye Center for Microbial Oceanography Research and Education, University of Hawai‘i at Mănoa, Honolulu, HI, United States

**Keywords:** planktonic Archaea, Marine Group I Archaea, Marine Group II Archaea, Thaumarchaeota, Thermoproteota, Thermoplasmatota, Poseidoniales, Nitrosopumilaceae

## Abstract

In 1977, Woese and Fox leveraged molecular phylogenetic analyses of ribosomal RNAs and identified a new microbial domain of life on Earth, the Archaebacteria (now known as Archaea). At the time of their discovery, only one archaebacterial group, the strictly anaerobic methanogens, was known. But soon, other phenotypically unrelated microbial isolates were shown to belong to the Archaea, many originating from extreme habitats, including extreme halophiles, extreme thermophiles, and thermoacidophiles. Since most Archaea seemed to inhabit extreme or strictly anoxic habitats, it came as a surprise in 1992 when two new lineages of archaea were reported to be abundant in oxygen rich, temperate marine coastal waters and the deep ocean. Since that time, studies of marine planktonic archaea have revealed many more surprises, including their unexpected ubiquity, unusual symbiotic associations, unpredicted physiologies and biogeochemistry, and global abundance. In this Perspective, early work conducted on marine planktonic Archaea by my lab group and others is discussed in terms of the relevant historical context, some of the original research motivations, and surprises and discoveries encountered along the way.

## Introduction

Members of the domain Archaea are now known to have diversified and radiated into a variety of disparate habitats in both aquatic freshwater, marine, and terrestrial environments. When the Archaea were first recognized *via* phylogenetic analyses of ribosomal RNA sequences of pure cultures (Woese and Fox, 1977; Woese, 1987), they appeared at first to be a narrowly distributed group found in only a few specific, often extreme habitats, including hydrothermal springs and vents, hypersaline ponds, and anoxic niches. Their considerable phylogenetic and habitat diversity, global abundance, and widespread distribution in marine plankton and elsewhere have recently been recognized over the past several decades (DeLong, 1998, 2003; Santoro et al., 2019; Baker et al., 2020). Ongoing discoveries of archaeal ubiquity that first occurred the 1990s were originally propelled by logical extensions of rRNA-based, cultivation-independent survey approach pioneered by Pace et al. in the mid-1980s (Pace et al., 1985, 1986; Pace, 1997).

The ubiquity of planktonic Archaea was first realized in the early 1990s, when two new groups of planktonic Archaea were initially reported. One new clade was peripherally related to the Crenarchaeota (DeLong, 1992; Fuhrman et al., 1992), and was dubbed Marine Group I Archaea (DeLong, 1992). Marine Group I Archaea have recently been proposed to be assigned to the new phylum Thermoproteota (Parks et al., 2020; Rinke et al., 2020). The other new archaeal clade (Marine Group II Archaea) was found to be affiliated with the euryarchaeotal Thermoplasma lineage (DeLong, 1992). Marine Group II have recently been assigned to the phylum Thermoplasmatota in the order Poseidoniales. Two phylotypes called Marine Group IIa and IIb Archaea (Massana et al., 2000) have been placed within the new families Ca. Poseidoniaceae and Ca. Thalassarchaeaceae, respectively (Rinke et al., 2019, 2020). Soon after the discovery of Group I and Group II planktonic Archaea, two other new lineages of planktonic Archaea were also discovered during cultivation independent surveys in marine environments (Fuhrman and Davis, 1997; López-García et al., 2001b).

Several excellent reviews and articles focussed on marine planktonic Archaea have recently been published (Stahl and de la Torre, 2012; Zhang et al., 2015; Haro-Moreno et al., 2017; Pereira et al., 2019; Santoro et al., 2019; Baker et al., 2020), so readers are referred to those for more comprehensive overviews. This short Perspective deliberately focuses on a brief historical account of own my lab’s adventures in studying marine planktonic Archaea over the past 30 years. The intent of this brief account is to provide some context and historical perspective on past, current, and ongoing work on marine planktonic Archaea (reported in this collection and elsewhere), as new discoveries about archaeal denizens of the oceans continue to emerge. Apologies in advance to the many excellent contributors to the marine archaeal field, whose work may not be cited herein due to the brevity of this Perspective.

## Planktonic Archaeal Discovery: A Falsified Hypothesis Produces a Surprising Finding

My lab’s first encounter with the marine planktonic Archaea originated in a research project funded by an Office of Naval Research Young Investigator Award while an Assistant Scientist at the Woods Hole Oceanographic Institution in 1989, and just as I began an Assistant Professorship at UC Santa Barbara in 1991. The goal of the hypothesis-driven research was to try to answer a longstanding question in marine science, namely: Does the ocean’s subsurface methane maximum originate from archaeal methanogens, that may live in anoxic microhabitats in the interior of particulate organic matter and marine snow? It was known at the time that oxygen rich near-surface ocean waters were supersaturated with considerable amounts of methane (Scranton and Brewer, 1977; Reeburgh, 2007). Yet the suspected producers of this methane were expected to be strictly anaerobic archaeal methanogens, which cannot thrive in oxygenated habitats (Liu and Whitman, 2000). So, what could be the source of all this methane in oxygen rich habitats? This conundrum was dubbed the Ocean Methane Paradox, since it was unclear then how strictly anaerobic processes like archaeal methanogenesis could produce large amounts of methane in oxygen rich ocean waters (Reeburgh, 2007; Karl et al., 2008; Repeta et al., 2016). The main proposed solutions to the paradox centered on the idea of a “false benthos,” e.g., anoxic niches in the water column, within marine snow particles or fish guts, for example, that could enable archaeal methanogenesis in otherwise well oxygenated habitats (Oremland, 1979; Sieburth, 1987; Kiene, 1991; Reeburgh, 2007).

So, the central goal of my ONR Young Investigator work was to test the hypothesis that archaeal methanogens were enriched in particle-associated, anoxic microhabitats in the ocean’s water column. These postulated anoxic micro-niches were proposed to enable methanogens to thrive, and generate the methane necessary for sustaining the subsurface methane maximum in the water column. As a corollary, I had postulated that Archaea should *not* be found as free-living planktonic cells in the water column (based on the archaeal phenotypes known at the time). To test these hypotheses, I applied archaeal-specific PCR primers to screen for archaeal DNA in coastal seawater and marine snow particle samples. PCR amplified archaeal rRNA sequences were then characterized by cloning and sequencing, and additionally group-specific, quantitative hybridization probe experiments of extracted ribosomal RNAs from the same samples (DeLong, 1992).

My results turned out to disprove the original hypothesis of predicted archaeal distribution patterns within plankton and marine snow particles: First, the data did not support the primary hypothesis, that archaeal methanogens were enriched in presumed anoxic microhabitats found in marine snow particles. Second, my results also refuted the secondary corollary, that Archaea should be *absent* in picoplankton assemblages of well-oxygenated, cold ocean waters. The data implied the opposite. Specifically, the results revealed that two new groups of planktonic Archaea (GI and GII Marine Archaea), were abundant free-living microbes in coastal seawaters (DeLong, 1992). More specifically, my analyses showed that: 1. The new Archaea were associated with small plankton size fractions, and not with marine snow particles; 2. Planktonic archaeal rRNAs represented appreciable amounts of total rRNAs (up to 2%) off the west and east coast of the United States; 3. Two archaeal clades were present in coastal samples, one loosely affiliated with the Crenarchaeota, and one specifically associated with the euryarchaeotal Thermoplasma lineage. 4. rRNA secondary structures and domain signatures diagnostic for Archaea were conserved within both new marine planktonic archaeal groups; and 5. The low GC content and greater phylogenetic distance of Group I Archaea from cultivated Archaea, complicated its precise phylogenetic placement solely on the basis of rRNA gene phylogeny (DeLong, 1992). At about the same time the above work was accepted for publication, a Nature paper describing the Marine Group I Archaea recovered from two subsurface seawater samples from 100 m and 500 m depth, collected 350 miles offshore San Diego, CA, United States was published (Fuhrman et al., 1992). As an aside, while Archaea did not appear abundant on marine snow particles (DeLong, 1992), there was a pronounced niche partitioning between particle-attached Bacteria on marine snow vs. those found free-living in surrounding waters (DeLong et al., 1993).

Surprisingly, it later turned out that there *is* a link between Marine Group I Archaea and the subsurface methane maximum – just not in the ways we had suspected! Specifically, my colleague David Karl had proposed that the subsurface methane maximum was not due to archaeal methanogenesis, but was rather the result of aerobic, bacterial utilization of methylphosphonates (as a source of phosphorus), which produces methane as a byproduct (Karl et al., 2008). In support of this postulate, Chon Martinez and Oscar Sosa in my lab showed that many marine bacteria encoded new as well as well-known phosphonate catabolic pathways, and that some were capable of aerobic methane production from methylphosphonates (Martinez et al., 2010, 2013; McSorley et al., 2012; Repeta et al., 2016; Sosa et al., 2017). Some had argued, however, that Karl’s postulated methane pathway was unlikely, since at the time there were no known sources of methylphosphonates in marine habitats. Somewhat ironically, those very same critics later published a paper showing that a marine Group I Archaea isolate (e.g., *Nitrosopumilus maritimus*), as well other planktonic bacteria, can produce methylphosphonates, and so potentially can provide the substrates necessary for bacterial aerobic methane production (Metcalf et al., 2012)! Recently, Repeta et al. (2016) definitively showed that naturally occurring marine dissolved organic matter contains abundant polysaccharide methylphosphonate esters that can fully account for the methane flux observed in the ocean’s subsurface methane maximum. While strictly anaerobic archaeal methanogenesis may not be the explanation for the marine methane maximum, marine planktonic Archaea had a role to play!

Higher order taxonomic designations tend to be fluid, as knowledge accumulates, and systematic nomenclatural rules change and evolve. In the past, the naming of bacterial and archaeal higher order taxa has been particularly problematic. Now, the recent availability of tens of thousands of new genome sequences, concatenated protein sequence phylogenies from conserved single copy genes, and new quantitative approaches to guide the assignment of higher order taxa, is leading to new phylum, class, order, and family designations in Bacteria and Archaea (Parks et al., 2018, 2020; Rinke et al., 2020). These genome-based approaches for assigning higher order taxon designations represent an improved and standardized approach that maintains internal consistency, as well as compliance with the Prokaryote Code rules (Garrity et al., 2019; Murray et al., 2020; Parks et al., 2020). As an example, in NCBI nomenclature, different Marine Group I Archaea are still labeled as belonging to either the Crenarchaeota or Thaumarchaeota. In the newly proposed genome-based nomenclatural system, Marine Group I Archaea are designated as members of the domain Archaea, phylum Thermoproteota, class Nitrososphaeria, order Nitrososphaerales, and family Nitrosopumilaceae (Parks et al., 2020; Rinke et al., 2020). Similarly, Marine Group IIa and IIb Archaea (Massana et al., 2000) have been subdivided into the families Poseidoniaceae and Thalassarchaeaceae, in the phylum Thermoplasmatota, class Poseidoniia, and order Poseidoniales (Rinke et al., 2019; Tully, 2019; Parks et al., 2020). In keeping with the times and improved systems of nomenclature, throughout the rest of this article I will refer to Marine Group I Archaea as planktonic Nitrosopumilaceae (also formerly called planktonic Crenarchaeota, then Thaumarchaeota), and to Marine Group II Archaea as planktonic Poseidoniales (also formerly called planktonic Euryarchaeota).

## Distribution and Abundance of Marine Archaea in the World Oceans

Following their initial recognition in 1992, my lab set out to map the distributions of planktonic Nitrosopumilaceae and Poseidoniales in a variety of marine habitats. One serendipitous surprise arose from Antarctic marine seawater samples that Rafaeal Jovine (then a graduate student at UC Santa Barbara in Barbara Prezlin’s lab) had shared with us to analyze. I did not expect to see much, but actually we found very high abundances of planktonic marine Archaea rRNAs in seawater from the frigid coastal waters off the Antarctic Peninsula (DeLong et al., 1994). Surprisingly, the Antarctic marine Archaea comprised a large proportion of this polar picoplankton community, up to 26% of the total community rRNA. Both the planktonic Nitrosopumilaceae and Poseidoniales clades were represented, whose rRNA sequences were highly similar to those found earlier in Santa Barbara and Woods Hole coastal waters (DeLong, 1992; DeLong et al., 1994).

**Figure 1:**
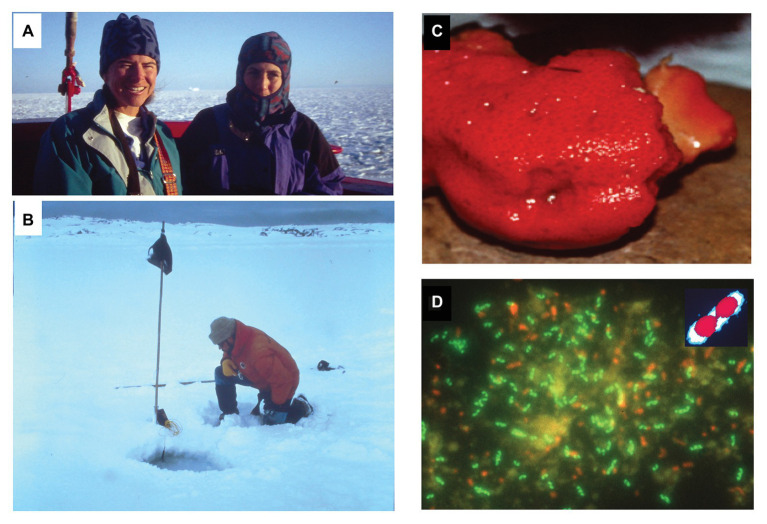
Exploring habitats where marine planktonic Nitrosopumilaceae and Poseidoniales are found. **(A)** Alison Murray (left) and Christina Preston (right) in transit to Palmer Station on our first trip to the Antarctic Peninsula. Pancake ice is visible in the background, as we near the Antarctic Peninsula in late austral winter, 1995. **(B)** The author staring at cold-water Archaea, and taking a temperature measurement at one of our sampling sites in a hole drilled through the sea ice, near Palmer Station Antarctica. **(C)** A specimen of the sponge *Axinella mexicana* found offshore the Santa Barbara coast, which harbors dense populations of the archaeon *Cenarchaeum symbiosum*. **(D)** Epiflourescent micrograph of sponge-derived *C. symbiosum* cells visualized with fluorescein-labeled rRNA-targeted oligonucleotide probes (DeLong et al., 1989). Many of the green-fluorescing *C. symbiosum* cells can be seen to be actively dividing. The inset shows a colorized version of one of the dividing *C. symbiosum* cells. The outer portion of the cell in the inset that is blue, shows where the cell was stained by fluorescein-labeled archaeal rRNA-targeted oligonucleotide probes. The red areas in the inset depict the area where the dye DAPI stained the chromosomal DNA. Note that there appears a nucleoid region (the DAPI-staining area in red in the inset) that occludes the fluorescein-labeled rRNA-targeted oligonucleotide probe binding. This corresponds to the central dark regions in the middle of individual *C. symbiosum* cells in the larger micrograph.

Expeditions to the Southern hemisphere soon followed to confirm these initial observations. Then graduate students Alison Murray, Christina Preston, and I set out in August 1995 in the late austral winter, to travel to Palmer Station Antarctica (Figure 1). Although we were beset in sea ice for 10 days after our first Drake Passage transit south, on another try, we eventually reached the Antarctic Peninsula in mid-September. The sea ice was so thick in late winter 1995, most of our seawater samples were pumped through holes drilled in the sea ice, collected into 50-liter carboys, and then hauled back to Palmer Station on sleds, cross-country skiing across the sea ice! Our studies at Palmer Station Antarctica during 1995 and 1996 showed high levels of marine Archaea throughout the Antarctic water column, with maxima in the upper 100 m (Murray et al., 1998). Additionally, much higher relative abundances of planktonic Nitrosopumilaceae were observed in the austral winter vs. summer (Murray et al., 1998). Although planktonic Nitrosopumilaceae had higher *relative* abundances in the austral winter, it was later shown that this is mostly attributable to large seasonal fluctuations in absolute numbers of planktonic bacteria in Antarctic waters, where bacterial cell densities can be 6-fold less during the austral winter compared to summertime (Church et al., 2003). In further work, Ramon Massana (then a postdoc in my lab) confirmed and extended similar Antarctic observations in the Gerlache Straight region. There, planktonic Nitrosopumilaceae were the most abundant group throughout the water column, while the less abundant planktonic Poseidoniales exhibited highest densities in surface waters (Massana et al., 1998). In her thesis work, Murray further showed evidence supporting a circumpolar distribution of Archaea *via* the Antarctic Circumpolar Current (Murray et al., 1999a). The planktonic Poseidoniales are now also known to occur in Arctic Ocean as well (Bano et al., 2004; Galand et al., 2009), in addition to Antarctic, temperate and tropical seas (Zhang et al., 2015; Santoro et al., 2019).

**Figure 2:**
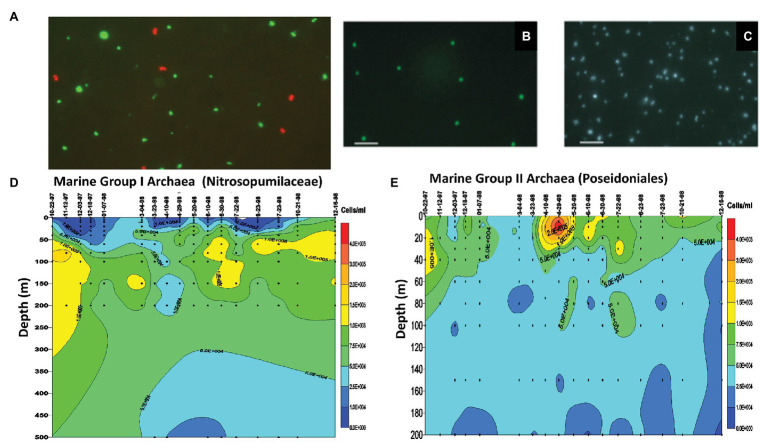
Single cell phylogenetic identification and enumeration of planktonic marine Archaea. **(A)** Marine Group I Archaea (planktonic Nitrosopumilaceae) from 80 m depth offshore Moss Landing, CA, United States. The red fluorescing cells are the Marine Group I Archaea (planktonic Nitrosopumilaceae) visualized with Texas Red-labeled rRNA-targeted polynucleotide probes. The green fluorescing cells are bacteria visualized with fluorescein-labeled bacterial probes (DeLong, 1999). **(B)** Marine Group II Archaea (planktonic Poseidoniales) from 80 m depth offshore Moss Landing, CA, United States. The green fluorescing cells are the Marine Group II Archaea (planktonic Poseidoniales) visualized with fluorescein-labeled rRNA-targeted polynucleotide probes. **(C)** Epifluorescence micrograph of all cells stained with DAPI, corresponding to the same microscopic field as visualized in panel **(B)**. **(D)** Time series measurements of Marine Group I Archaea (planktonic Nitrosopumilaceae) cell densities from depth profiles taken at mooring station M1 off Moss Landing, CA, United States. After Mincer et al. (2007). **(E)** Time series measurements of Marine Group II Archaea (planktonic Poseidoniales) cell densities from depth profiles taken at mooring station M1 off Moss Landing, CA, United States. After Mincer et al. (2007).

In parallel with Antarctic studies in the late 1990s and early 2000s, we also surveyed planktonic Nitrosopumilaceae and Poseidoniales in temperate and subtropical waters of the North Pacific. In the Santa Barbara Channel, we showed that total archaeal rRNA abundances were lower in surface waters and reached highest values (as much as 30% of the total prokaryotic rRNA) at depths below 100 m (Massana et al., 1997). Quantitative rRNA hybridization experiments indicated that while planktonic Nitrosopumilaceae were most abundant at depth, the planktonic Poseidoniales exhibited maximum relative abundances in surface waters (Massana et al., 1997). These same trends were also reflected in the relative recoveries of planktonic Nitrosopumilaceae and Poseidoniales in rRNA gene clones at in surface vs. deep waters. Extending these results, we observed spring and summertime “blooms” of planktonic Poseidoniales in surface waters over a 3-year time series in the Santa Barbara Channel (Murray et al., 1999b). In the same study, deeper waters exhibited temporally even distributions and higher abundances of planktonic Nitrosopumilaceae (Murray et al., 1999b). Positive correlations between water column nitrite concentrations and planktonic Nitrosopumilaceae were also observed in early studies (Murray et al., 1999b), a result now more readily interpreted since the first cultivation an affiliate of Marine Group I Archaea, the ammonia-oxidizing archaeon *N. maritimus*, by Dave Stahl and his group (Konneke et al., 2005). A bit later, we used single-cell enumeration by *in situ* hybridization employing high-sensitivity, fluorescently labeled polynucleotide probes (DeLong et al., 1999), which provided cellular abundance profiles and time series measurements of planktonic Nitrosopumilaceae and Poseidoniales in the Monterey Bay, the North Pacific Subtropical Gyre, and Antarctica (Figure 2; DeLong et al., 1999; Karner et al., 2001; Church et al., 2003; Mincer et al., 2007). Over the years, these results have been extended considerably, and it is now known that different lineages of planktonic Poseidoniales are found at different depths and times in the water column in temperate, subtropical, and polar seas alike (Massana et al., 2000; López-García et al., 2001a,b; DeLong et al., 2006; Frigaard et al., 2006; Galand et al., 2009; Martin-Cuadrado et al., 2015; Zhang et al., 2015; Santoro et al., 2019).

In coastal surface waters in spring and summer, planktonic Poseidoniales are sometimes observed to bloom, reaching high densities (Murray et al., 1999b; Pernthaler et al., 2002; Mincer et al., 2007).

Early results indicated that planktonic Archaea were ubiquitous and abundant in the oceans, and were biogeochemically significant – but in unknown ways at the time. Conclusions from early field work of 20 years ago do appear to have held up to the test of time, for example: “The distribution of the 2 archaeal groups [*e.g., planktonic Nitrosopumilaceae and Poseidoniales*] suggested that they responded independently to environmental conditions, are physiologically different, and likely participate in different environmental processes” (Murray et al., 1999b); and “An alternative hypothesis is that the archaea [e.g., planktonic *Nitrosopumilaceae*] are chemoautotrophs that grow slowly but are not affected by the low-carbon conditions prevalent in the austral winter” (Murray et al., 1998). Years later, the fortuitous cultivation of the ammonia-oxidizing isolate *N. maritimus* (Konneke et al., 2005), along with comparative genome sequence analyses of *Cenarchaeum symbiosum* (Hallam et al., 2006b) added to our current knowledge and perspective of planktonic marine archaeal physiology ecology and evolution – especially for the planktonic Nitrosopumilaceae.

## *Cenarchaeum Symbiosum*: Some Coastal Nitrosopumilaceae are Symbionts of Sponges

During her thesis work in my lab at UC Santa Barbara, Christina Preston was studying the diversity of bacterial assemblages in marine sponges. Chris told me she was going to try out the archaeal PCR primers to find archaea in sponge microbial assemblages. At the time, I told Chris “Sure, go for it (but you probably will not find anything).” Once again, DeLong was incorrect, and this time in a big way! (Note to students: Question authority, but make sure to back it up with data eventually – as Chris did!).

In her thesis work, Chris Preston showed that one of the sponge species we were examining (Figure 1; *Axinella mexicana*), harbored an extraordinarily abundant population of marine Archaea, often equaling or exceeding co-occurring bacterial abundances in *A. mexicana* individuals (Preston et al., 1996). She further showed that these unanticipated sponge Archaea were affiliated with marine Nitrosopumilaceae, and were specifically related to, but distinct from, other known planktonic Nitrosopumilaceae. All *A. mexicana* specimens collected at different times and at different sites yielded just one main archaeal rRNA phylotype, that of *C. symbiosum* (Preston et al., 1996). Since no other sponges sampled in the same habitat harbored *C. symbiosum* species, and all *A. mexicana* indivduals did, it became clear that *C. symbiosum* was a true *A. mexicana* symbiont. Fluorescently labeled rRNA probe experiments showed that a large proportion of *C. symbiosum* cells in sponge host tissues were actively dividing at 10°C (Figure 1), conclusively demonstrating that these archaeal relatives of thermophiles thrived at low temperatures within sponge tissues (Preston et al., 1996).

*Axinella mexicana* provided in essence a pure cell enrichment of *C. symbiosum*, so with these and some Antarctic picoplankton cell preparations, we were able to identify and link specific archaeal tetraether lipids with the newly discovered planktonic Nitrosopumilaceae (DeLong et al., 1998). Additionally, using samples of *C. symbiosum* that we had sent him, Jaap Damsté and colleagues identified a new archaeal lipid, crenarchaeol, that could be linked specifically to the planktonic Nitrosopumilaceae lineage (Damsté et al., 2002). Since our early work on these archaeal sponge symbionts, *C. symbiosum* relatives have been reported in other sponges (Garcia-Bonilla et al., 2019; and references therein) as well. In addition, free-living relatives of *C. symbiosum* were recently isolated in seawater ammonia-oxidizer enrichments originating from Hood Canal, Washington (Qin et al., 2020).

## Molecular Biology and Genomics of Planktonic Marine Archaea: The Early Days

At about the same time *C. symbiosum* was discovered, my lab was actively developing large DNA fragment clone libraries for genomic analyses of environmental samples. Our interest stemmed from my earlier lambda clone library construction efforts in Norm Pace’s lab (Schmidt et al., 1991), and fruitful collaborations we had with Hiroaki Shizuya and Jeff Stein in Mel Simon’s group. With these colleagues, we leveraged high fidelity fosmid clone libraries, a technology derived from Bacterial Artificial Chromosome vectors, developed for use in the human genome project (Kim et al., 1996). We prepared fosmid libraries from planktonic microbial community DNA, to study planktonic Nitrosopumilaceae using a gene linkage-based, “chromosomal walking” approach. Cloning and analyses of large genome fragments from planktonic Nitrosopumilaceae confirmed the phylogenetic placement of planktonic Nitrosopumilaceae using alternative highly conserved protein sequences, including the elongation factor EF2 (Stein et al., 1996). Using similar approaches, Oded Béjà in my group at MBARI later showed that co-existing Antarctic planktonic Nitrosopumilaceae populations harbored considerable genomic microdiversity but also shared considerable gene synteny with the *C. symbiosum* genome (Béjà et al., 2002).

Fortuitously, the availability of near pure preparations *C. symbiosum* cells facilitated some deeper understandings about marine Nitrosopumilaceae. For example, using *C. symbiosum* and Antarctic picoplankton cell preparations, we were able to directly link archaeal tetraether lipids usually found in thermophiles, with cold-dwelling planktonic Nitrosopumilaceae (DeLong et al., 1998). Christa Schleper, then a postdoc in the lab, developed methods to purify *C. symbiosum* cells on Percoll gradients, to construct large fragment *C. symbiosum* genome libraries. In a functional metagenomic study, Christa showed that *C. symbiosum* encoded a family B (a-type) DNA polymerase that shared greatest amino acid identity with homologues from the thermophilic Thermoproteota *Sulfolobus solfataricus* (Schleper et al., 1997, 1998). Further, the *C. symbiosum* DNA polymerase was heterologously expressed in *Escherichia coli*, purified, and analyzed to determine its activity and biochemical characteristics. Christa showed that the *C. symbiosum* DNA polymerase was rapidly inactivated at temperatures above 40°C, and was much more thermolabile than DNA polymerases from its thermophilic relatives, in keeping with *C. symbiosum*’s cold water origins (Schleper et al., 1997). Christa’s work went on to show that *C. symbiosum* exists in *A. mexicana* as a population of at least two very highly related but distinguishable strain variants, as opposed to being a completely clonal population (Schleper et al., 1998).

Arguably, the field of metagenomics started with these early large fragment DNA environmental cloning and sequencing experiments, as originally envisioned by Pace et al. (Pace et al., 1985, 1986; Olsen et al., 1986), that were first instantiated in environmental genomic studies of marine picoplankton and Archaea (Schmidt et al., 1991; Stein et al., 1996; Schleper et al., 1997, 1998). After these ideas of cultivation-independent microbial community genomics had been proposed, tested, and proven as described above, the term “metagenomics” was later coined and popularized (Handelsman et al., 1998). The advent of high throughput shotgun cloning and sequencing, combined with next generation sequencing approaches inspired by the human genome project, eventually led to pioneering new work (Tyson et al., 2004) that typifies much of the currently blossoming phase of cultivation-independent microbial genomics (aka metagenomics) of today.

Similar genome-enabled approaches also helped shed some light on physiological properties of planktonic Poseidoniales as well (Frigaard et al., 2006). While genome gazing at fosmid library sequence data from the North Pacific Subtropical Gyre, I had noticed something unusual. Specifically, I found that some photoprotein genes of proteorhodopsins (that we had originally discovered in members of the domain Bacteria; Béjà et al., 2000) were associated with planktonic Poseidoniales genome fragments as well. Niels-Ulrik Frigaard, then a postdoc in my lab at MIT, extended this preliminary observation with some very nice analyses that showed that: 1. About 10% of planktonic Poseidoniales in the photic zone contained a proteorhodopsin gene adjacent to their SSU rRNA; 2. Planktonic Poseidoniales proteorhodopsins were found within other genomic regions as well; and 3. Proteorhodopsin-containing Poseidoniales were found only in the photic zone, even though Poseidoniales were found in considerable numbers at depth (Frigaard et al., 2006). Our analyses suggested that proteorhodopsins confer significant light-dependent fitness contributions, which in part explains their inter-domain lateral gene transfer, acquisition, and retention, and their high abundance in the photic zone (Frigaard et al., 2006; Pinhassi et al., 2016). Our observations were later confirmed when the first planktonic Poseidoniales genome sequence was reported (Iverson et al., 2012).

Genomic analysis of *C. symbiosum* eventually provided the first available genome sequence for planktonic Nitrosopumilaceae (Hallam et al., 2006a). The *C. symbiosum* project team, led by Steve Hallam and myself, included past and current postdocs from my lab (Steve Hallam, Kostas Konstantinidis, Christa Schleper, José de la Torre, Christina Preston), and along with other colleagues, we reported on the properties of the complete genomic complement of *C. symbiosum* (Hallam et al., 2006a). Most all of the expected archaeal core genes were present in the *C. symbiosum* genome. In addition to core genome properties, archaeal “house keeping” genes, energy metabolism genes (including those involved in ammonia oxidation), CO_2_ assimilation pathways, and some new expanded gene families were reported (Hallam et al., 2006a). In the *C. symbiosum* ammonia oxidation pathway, in addition to ammonia monooxygenase and ammonia permease, urease and a urea transport system were also present. Some of the new genes found in *C. symbiosum* were speculated to be involved with the archaeal-sponge symbiotic association. Analyses of *C. symbiosum* universally conserved concatenated protein phylogenies, confirmed its deeply branching, peripheral relationship to the Crenarchaeota originally inferred from rRNA phylogenies. The *C. symbiosum* genome also proved useful in environmental studies leveraging genome fragment recruitment, as well as for early analyses of planktonic Nitrosopumilaceae energy and carbon assimilation pathways (Hallam et al., 2006b). In addition to earlier elongation factor phylogenies (Stein et al., 1996) and DNA polymerase structure-function work (Schleper et al., 1997), analyses of *C. symbiosum* histones and recombination proteins (Rad A) also provided new insights into the relationship between planktonic Nitrosopumilaceae and other cultivated Archaea (Sandler et al., 1999; Cubonova et al., 2005). After the *C. symbiosum* genome was published in 2006, genomic analyses of the first ammonia-oxidizing planktonic Nitrosopumilaceae isolate, *N. maritimus* provided important additional perspective on the physiological properties and biogeochemistry of planktonic Nitrosopumilaceae (Walker et al., 2010).

## Where Next, Marine Archaea?: Mag Explosions, Culture Revolutions, and Going Viral

There is certainly much more to learn about currently known marine planktonic archaeal groups. New planktonic Archaeal lineages undoubtedly remain to be discovered and described *via* both cultivation-dependent and cultivation-independent approaches. As one new example, it is becoming clear that members of the ubiquitous Woesearchaeota lineage are likely regular residents of marine plankton assemblages, and are not just found at hydrothermal vents (Liu et al., 2018; Peoples et al., 2018). If and how they might be parasitically or symbiotically associated with other planktonic marine Archaea is an interesting question for the future. With respect to the physiology, biochemistry, and metabolism of diverse planktonic marine Archaea, heterologous expression and subsequent biochemical characterization of proteins and enzymes from planktonic marine Archaea still represents another very useful line of research to pursue (Schleper et al., 1997; Béjà et al., 2000; Martinez et al., 2007, 2010; McSorley et al., 2012; Michalska et al., 2015).

The explosion of newly available metagenome assembled genomes is providing much valuable new perspective (Parks et al., 2017, 2018, 2020). For example, even in the most well characterized group, the planktonic marine Nitrosopumilaceae (marine Group I Archaea), new evidence suggests that some lineages may be heterotrophic, instead of strict chemolithoautotrophs growing on ammonia (or urea) and CO_2_ (Aylward and Santoro, 2020; Reji and Francis, 2020). Within the planktonic Poseidoniales, new genome-enabled insights have led to the proposed subdivision of Marine Group IIa and IIb Archaea (Massana et al., 2000), into the families Poseidoniaceae and Thalassarchaeaceae, respectively (Rinke et al., 2019). Based on genomic information, members of the genus *Poseidonia* were inferred to be motile, aerobic, photoheterotrophic, and able to ferment a wide range of carbohydrates, in particular peptides (Rinke et al., 2019; Tully, 2019). In contrast, genomes from members of the genus *Thalassarchaeum* have suggested that they are non-motile aerobic heterotrophs, encompassing different species that can be either phototrophic or non-phototrophic (Rinke et al., 2019; Tully, 2019). New genome-enabled insights are also coming to light for Marine Group III Archaea, which like their close relatives, sometimes occur in surface waters and are capable of proteorhodopsin-based photoheterotrophy (Haro-Moreno et al., 2017).

A vexing question for Poseidoniales biology, ecology, and biogeochemistry that still remains to be answered is: What are the characteristic membrane lipids of this euryarchaeal group? Earlier lipid analyses of *C. symbiosum* and *N. maritimus* had clarified the tetraether lipid compositions, and presence of crenarchaeol, in members of marine planktonic Nitrosopumilaceae (DeLong et al., 1998; Damsté et al., 2002; Schouten et al., 2008). Yet to date, the membrane lipid composition of marine planktonic Poseidoniales remains virually unknown, and is still somewhat controversial (DeLong, 2006; Lincoln et al., 2014a,b; Wang et al., 2015; Besseling et al., 2020; Ma et al., 2020). The mysterious Marine Group IV Archaea, peripherally associated with the Haloarchaea (López-García et al., 2001b), are among the least well known among planktonic Archaea. Metagenomic-based genomic analyses have just recently provided new perspective on this group (now dubbed Hikarchaeia), linking their genomic properties and physiological potential, with the complex evolutionary transition from methanogens to extreme halophiles (Martijn et al., 2020).

Cultivation-based approaches will necessarily continue to provide critical new insights into the physiology and metabolism of marine planktonic Archaea. Fantastic successes, including cultivation of predominant oligotrophic marine bacterioplankton (Rappe et al., 2002), and cultivation of the first ammonia-oxidizing archaeon *Nitrosopumilus maritimus* (Konneke et al., 2005), represent classic examples. Notably however, currently available cultivation strategies do not enrich specifically for particular phylogenetic linages (albeit the use of specific antibiotics can be leveraged). For example, efforts of Stahl et al. originally deployed enrichments for ammonia-oxidizers, not for Archaea – but fortuitously, it just so happened that some Archaea turned out to be nitrifiers (Konneke et al., 2005)! In another spectacular example, the recent cultivation of Ca. *Prometheoarchaeum syntrophicum*, a representative of the Asgard Archaea (Imachi et al., 2020), represents a tour-de-force with far reaching implications that present exciting new research opportunities. But the sobering fact is that these successful archaeal enrichments took over a decade in the making, requiring careful, painstaking, dedicated work, before yielding their final fruit (Imachi et al., 2020). In the case of planktonic Archaea, to date, no cultivated representative of the marine Poseidoniales has yet been reported, representing an important challenge to the field. Certainly, plenty of room remains to improve and invent new, more efficient microbial cultivation strategies, including methods for massively parallel variation of culture conditions, improved cultivation and characterization strategies using low culture volumes and cell densities, as well as enrichment and metabolic dissection of microbial consortia and co-cultures.

Marine archaeal viruses and other mobile elements represent another emerging area for marine planktonic archaeal research (Krupovic et al., 2018). New reports on the nature, structure, and distribution of marine Nitrosopumilaceae viruses, proviruses, and mobile elements are appearing more and more frequently, providing new insight into their viral diversity, viral-host interactions, and lateral gene transfer (Krupovic et al., 2011; Ahlgren et al., 2019; Kim et al., 2019; Krupovic et al., 2019; Lopez-Perez et al., 2019; Luo et al., 2020). Less information is available on marine Poseidoniales viruses, also representing new opportunities for planktonic Archaea virus research (Philosof et al., 2017; Krupovic et al., 2018; Luo et al., 2020). Leveraging metagenomics, metatrascriptomics, single-cell metagenomics, and of course the eventual cultivation of planktonic Poseidoniales representatives, will help to push progress in this area.

The general area of marine Archaea research is in a very vigorous phase currently, and continues to gather steam, generating new genomic, biochemical, biogeochemical, evolutionary and ecological data, and hypotheses. This in turn informs the growing appreciation of the extent of archaeal habitat distributions, archaeal physiological and genomic diversity, archaeal ecological significance and their evolutionary relationships with eukaryotes. As an example, recent analyses of the nature and evolution of the core archaeal pathways of methane and short chain hydrocarbon metabolism have provided important new evolutionary and ecological insights into the evolution of carbon metabolism in the Archaea (Evans et al., 2019; Seitz et al., 2019; Wang et al., 2019). Parallel analyses of marine planktonic Archaea, to further assess their core genomic, pan-genomic, and metabolic repertoires, their viruses and mobile elements, and their environmental and community interactions are anticipated. The resulting deeper understandings of archaeal ecology, biogeochemistry, and evolution will continue to propel planktonic marine Archaea research well into the future.

## Data Availability Statement

The original contributions presented in the study are included in the article/supplementary material and further inquiries can be directed to the corresponding author.

## Ethics Statement

Written informed consent was obtained from the individual(s) for the publication of any potentially identifiable images or data included in this article.

## Author Contributions

The author confirms being the sole contributor of this work and has approved it for publication.

### Conflict of Interest

The author declares that the research was conducted in the absence of any commercial or financial relationships that could be construed as a potential conflict of interest.

The handling Editor declared a past co-authorship with one of the author ED.
